# Global health partnerships: building multi-national collaborations to achieve lasting improvements in maternal and neonatal health

**DOI:** 10.1186/s12992-016-0159-7

**Published:** 2016-05-20

**Authors:** Rohit Ramaswamy, Brianne Kallam, Dragica Kopic, Borislava Pujic, Medge D. Owen

**Affiliations:** Public Health Leadership Program, Gillings School of Global Public Health, University of North Carolina, 4107, McGavran-Greenberg Hall, Chapel Hill, NC USA; Kybele, Inc., Winston-Salem, NC USA; Department of Anesthesiology, University Hospital, Split, Croatia; Department of Anesthesiology, Clinical Center Volvodina, NoviSad, Serbia; Department of Anesthesiology, Wake Forest School of Medicine, Winston-Salem, NC USA

**Keywords:** Global health partnerships, Low and middle income countries, Maternal and child health, Obstetric anesthesia

## Abstract

**Background:**

In response to health care challenges worldwide, extensive funding has been channeled to the world’s most vulnerable health systems. Funding alone is not sufficient to address the complex issues and challenges plaguing these health systems. To see lasting improvement in maternal and infant health outcomes in the developing world, a global commitment to the sharing of knowledge and resources through international partnerships is critical. But partnerships that merely introduce western medical techniques and protocols to low resource settings, without heeding the local contexts, are misguided and unsustainable. Forming partnerships with mutual respect, shared vision, and collaborative effort is needed to ensure that all parties, irrespective of whether they belong to resource rich or resource poor settings, learn from each other so that meaningful and sustained system strengthening can take place.

**Methods:**

In this paper, we describe the partnership building model of an international NGO, Kybele, which is committed to achieving childbirth safety through sustained partnerships in low resource settings. The Kybele model adapts generic stages of successful partnerships documented in the literature to four principles relevant to Kybele’s work. A multiple-case study approach is used to demonstrate how the model is applied in different country settings.

**Results:**

The four principle of Kybele’s partnership model are robust drivers of successful partnerships in diverse country settings.

**Conclusions:**

Much has been written about the need for multi-country partnerships to achieve sustainable outcomes in global health, but few papers in the literature describe how this has been achieved in practice. A strong champion, support and engagement of stakeholders, co-creation of solutions with partners, and involvement of partners in the delivery of solutions are all requirements for successful and sustained partnerships.

## Background

Progress has been made in reducing maternal and newborn mortality worldwide, but Millennium Development Goals 4 and 5 will not be met. Disparity in maternal and infant health outcomes remains stark between low and high income countries. Unique challenges within each country eliminate a one-size fits all approach and demand creative solutions to address local healthcare problems. These solutions need to go beyond mere clinical training and necessitate the strengthening of systems to improve the quality of care and patient satisfaction. These complex interventions cannot be achieved without the customization of global best practice in collaboration with local care providers. Collaborative quality improvement initiatives in healthcare are described in the US and UK; however, few have been reported in low and middle income countries (LMIC) [1]. The nature of these collaborations, especially across resource gaps, must go past the good intentions that drive humanitarian assistance programs, since without genuine participation from host countries, intended collaborations can devolve into tokenism [2]. Authentic local partner participation and willingness from both parties to learn together can yield innovative, lasting results. In this paper, the tenets of productive global health partnerships will be explored, and a multi-country partnership approach developed by a US based non-governmental organization, Kybele, will be described.

### Overview of Kybele

Kybele (www.kybeleworldwide.org) is a volunteer driven, 501(c)(3) humanitarian organization dedicated to improving childbirth safety through innovative partnerships in low resource settings. Kybele’s mission is to institute collaborative programs in obstetric, anesthesia and pediatric departments within LMIC tertiary hospitals to improve the quality of maternal and newborn care through the strengthening of clinical practices and service delivery processes. This includes care of the high-risk pregnant patient, administration of spinal, epidural, and general anesthesia, pain relief for childbirth, postoperative pain management, laparoscopic surgery techniques, ultrasound diagnosis, neonatal resuscitation, and advanced newborn care. In addition, Kybele reinforces these skills with operational capacity building, such as leadership development, quality improvement, clinical guideline development, standard operating procedures, advocacy, and monitoring and evaluation.. Kybele has worked in countries as diverse as Turkey, Croatia, Georgia, Armenia, Ghana, Brazil, Serbia, Mongolia, Egypt, Romania, and Vietnam..Kybele team members consist of anesthesiologists, obstetricians, nurses, midwifes, neonatologists, public health specialists, leadership consultants, equipment technicians, internists, and others.

Kybele’s focus is to strengthen the clinical capacities of medical professionals to care for healthy mothers and babies, and those at risk of complications. The particular combination of clinical and system strengthening activities differ by country which is why the partnership model is a fundamental component of Kybele’s approach. The topics that are most important, the manner in which these topics are addressed, the relative roles of the Kybele team members (vis-à-vis the local country team), the balance between clinical training and systems strengthening are all determined collaboratively and are based on a joint discussion of needs and priorities. As a partnership evolves, the focus shifts to ways of improving country-wide standards, not just conditions within selected hospitals through the creation of local networks.

### What makes a good partnership?

A summary of the literature outlines the following stages for successful partnership formation: 1) recognizing and accepting the need for partnership; 2) developing clarity and realism of purpose; 3) ensuring commitment and ownership; 4) developing and maintaining trust; 5) creating robust and clear partnership working arrangements, and 6) monitoring, measuring, and learning [3]. The literature emphasizes the importance of investing the necessary time and effort into developing a strong foundation to secure positive partnership outcomes. Partnerships do not form overnight. These elements are outlined in Table 1.

**Table 1:** Comparing Kybele’s partnership model to the stages of successful partnership

Stages of Successful Partnership	Kybele’s Model
Recognizing and accepting the need for partnership	Ensure that a champion is selected who is committed to a partnership
Develop clarity and reality of purpose	Develop local solutions based on assessment of need and capacity
Ensuring commitment and ownership	Obtain broader stakeholder support and negotiate cost sharing
Developing and maintaining trust	Commit to long term partner involvement by including them as future members of the Kybele team.
Robust and clear working arrangements	Kybele team leader plans for each visit and determines the best team composition to meet current priorities
Monitoring, measuring and learning	M&E integrated into all Kybele programs

A robust start is important, but partnerships also need to work well over time. Howell describes four key components to making global health programs effective and sustainable; engaged local champions; teaching and supporting appropriate concepts with cultural sensitivity; knowledgeable and adaptable team members; and continual follow-up with reinforcement and encouragement for local practitioners [4]. USAID emphasizes the importance of a shared vision between partners, stating that, “the most effective partnerships are those in which risks, responsibilities and rewards are shared, and which address core interests of all parties involved [5].” Similarly, the senior director of strategic partnerships and alliances at CARE states, “It’s really important to spend as much time as needed to ensure there is a shared vision, not just a one-year commitment but an opportunity to grow and leverage the skills each partner is uniquely positioned to bring to the table [6].”

It is not surprising that elements of the stages and characteristics of successful partnerships are common across sectors of health, business, politics, and information technology and have been reiterated by a variety of public, private, and non-profit entities. This indicates that foundational elements are necessary for global partnerships to be successful. However, in order to create and sustain successful partnership in practice, these elements need to be adapted to the context of the program and the kind of partners involved. Understanding how partnerships are developed in specific settings, and the lessons learned through this process, bridges the gap between theory and practice. In this paper, we describe how Kybele’s partnership model was developed and applied in various settings to incorporate these generic principles into its global programs. We end with some concrete guiding principles for developing successful multi-country global health partnerships.

### The Kybele partnership model

**Fig. 1:**
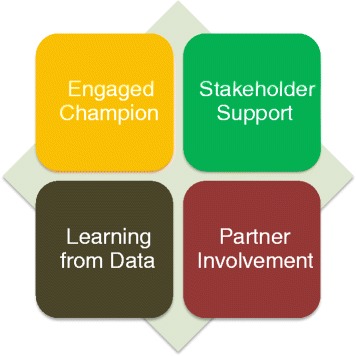
The figure shows the principles underlying Kybele’s partnership model

The Kybele partnership model described in this paper evolved over time and is based on four key principles, which are aligned with the six stages of partnership described earlier. In Table 1, we have translated the six steps into language relevant to the Kybele program context. Figure 1 shows the four principles of our model.

The first stage in successful partnerships described in the literature is *recognizing and accepting the need for a partnershi*p, and is also the critical first principle in the Kybele partnership model of Fig. 1. This needs to take place early, preferably during the first exploratory site visit, and the practical application of this takes place through the identification of individuals who are willing to serve as local champions, and the appointment of a Kybele team leader. Therefore, the need for partnership is reinterpreted in the Kybele model as the selection of an *engaged champion* without whom no partnership is possible. These individuals are pivotal to program success and will be involved in every element of program planning and execution. A local champion provides knowledge on local requirements, medical licensure, bureaucracy, legal issues, and culture, and lays the groundwork for the program to be successful and well-received [3, 7]. Typically, the best champions are mid-career professionals, senior enough to command authority, but are not yet close to retirement.

The next stage recommended in the review of the literature is to *develop clarity and reality of purpose* but in the settings that Kybele works in, this can be done only after there is greater commitment and ownership on the part of the local staff. Therefore the third and fourth stages in Table 1, *ensuring commitment and ownership* and *developing and maintaining trust* are part of the next relevant principle, *obtain stakeholder support* in the Kybele model. Kybele’s method for doing this is to engage a larger number of stakeholders in the program design process, be they hospital administrators, professional organizations, or the government health system. The champions play critical roles in this step by communicating project goals and objectives with other local stakeholders. They increase local buy-in by networking with colleagues to select appropriate hospital sites, and by facilitating agreements for tangible and demonstrable support for the partnership, for example by getting the stakeholders to bear some of the program costs such as accommodation, food, and local transport for Kybele team members during their visits, or of making facilities available for training [8]. In addition, groundwork is laid to explicitly establish the relationship between Kybele and its partner organizations as a long term enterprise, with trust being built by partners co-teaching, applying for grants, presenting at conferences and publishing papers with Kybele.

Once the champion and the stakeholders are in place, Kybele works with them to jointly *create robust and clear working arrangements* and to *develop and maintain trust*. These stages define the third Kybele partnership principle, *partner involvement*. Kybele does this by developing local, customized improvement plans that balance facility priorities with existing resources, through discussions with the champion and stakeholders. Where health systems are weak, the action plan may focus on an integrated capacity building approach aimed at clinical, operational, and leadership strengthening. Where systems are stronger, the focus may be primarily on filling clinical gaps. It is important to emphasize the fact that the distinct roles of the international and local partners are both critical for the success of the partnership. The international partners bring in “global” skills, but these are useless unless they are tested in the local context. Therefore while the partnership may not be “equal” in the sense that there is trainer/trainee relationship between the visiting and the local teams, it is impossible to be successful without the specific contribution of each partner.

Kybele’s on-site visits and face-to-face training are critical activities to build and maintain relationships and trust. Critics will argue that face-to-face mentored instruction between international partners is not sustainable [9]. However, as is extensively documented in the implementation science literature, training without support is a waste of time and does not produce sustainable results [10, 11]. This need for support is even more critical in resource-poor settings. The Kybele partnership model drives partner involvement through sustained face-to-face activity for several years. Without this activity, the local customization and joint planning and the continued building of trust that is crucial for success cannot take place.

*Monitoring, measuring and learning*, using both qualitative and quantitative data is built into all Kybele partnerships and is the fourth principle in Fig. 1. All projects collect data on both clinical and process outcomes and to use these data for planning the next set of activities. Data collection sheets, indicators, and planning for data collection is done collaboratively with partners to encourage transparency and accuracy.

## Methods

We have adopted a case study approach to document our experience of applying the Kybele partnership model. Our case study methodology is a practical version based on the research methods proposed by Yin [12] and by Stake [13]. Our research question has been to gain a deeper understanding of how the partnership model works in various contexts, and what aspects of the model should be emphasized in different settings, and is therefore appropriate for the use of a case study technique. The unit of analysis is the application of the Kybele model to an individual country, and we have adopted a multiple-case design where each country is its own case. This is an appropriate design because each country has its own context [12].

Our approach to data collection follows the approach of Stake [13] that “knowledge is constructed, rather than discovered”. Our data was collected over multiple years during the implementation of the programs in the various countries, and was discussed collaboratively with the partners in order to improve the strength of the partnerships and the quality of the outcomes. The complexity of Kybele’s work and the wide range of circumstances under which Kybele programs operate necessitated an iterative process of model development, based on experimentation and learning in different countries. Our data was assembled using three processes, though the timing and intensity of these processes differed by country and by case. We labeled the three processes employed for data collection as inquiry, observation, and reflection. In each country, data was typically collected during the Kybele team’s visit. *Inquiry* processes used traditional qualitative methods such as interviews, focus groups, and staff and patient surveys as well as quantitative data on deliveries, caesarian rates and the use of regional anesthesia techniques to understand whether the partnership was working and resulting in appropriate outcomes for mothers in the various countries. For the first visit in any country or facility, an assessment tool created to evaluate readiness for engaging in a partnership with Kybele was used. The methods for collecting data on subsequent visits varied by country, since the trajectory of each partnership evolved differently over time. *Observational* processes involved both clinical observations by members of the Kybele team, as well as observations on team dynamics, leadership behaviors and staff interactions where systems strengthening was the major priority. Observational data was collected using checklists that were refined and validated through discussions with the Kybele team and the partners. *Reflective* processes involved informal debrief sessions conducted at the end of each day by members of the Kybele team, as well as formal review sessions conducted by staff after major incidents such as a maternal death.

The primary responsibility for assembling, packaging and disseminating data rested with the Kybele *team leaders*, each of whom was also responsible for Kybele program development and growing stakeholder partnerships in one country. These team leaders were selected through an application process where references, qualifications, and medical licensure were screened, and an assessment was made of their diplomacy, maturity, and the ability to work effectively with others in an interdisciplinary team. Team leaders organized visits to their countries one or more times a year and sent detailed trip reports to the Kybele organizational staff. These trip reports served as an ongoing documentation of challenges, opportunities, and ideas for improvement, and were used to develop ongoing best practices for engagement and partnerships to achieve successful outcomes. In addition, team leaders, team members, and Kybele’s organizational staff came together every two years for a weekend summit, where data from multiple countries was shared and practices, processes, and tools to improve Kybele’s operations were discussed. These workshops provided additional opportunities to refine and improve Kybele’s partnership model.

To synthesize the results from the application of the model to various countries, we categorized the recorded data from each country according to the steps in Fig. 1 and highlighted the most salient principle in each country’s model to describe the implementation of the Kybele model in that country. We also aggregated our learning across countries by each principle of the Kybele model. This allowed us to focus on the individual countries in some detail while also exploring the common themes across countries.

## Results

It must be emphasized that our experience with the model presented in this paper continues to evolve, but the major elements are now in place. This process of developing a customized partnerships model is important not only because it establishes successful guidelines for practice but also because it builds the shared values and organizational culture within Kybele that are needed for the organization to function effectively as a loose federation of independent teams across multiple countries.

### Using the Kybele partnership model: country examples

The following country examples describe different aspects of the application of the partnership model in several countries. Both principles that contributed to successful partnerships in each country and those that failed in an instance of an unsuccessful partnership are highlighted.

#### Croatia

##### Engaged champion

In 2004, Kybele was invited to make a presentation on neonatal resuscitation at a university hospital in Croatia, an Eastern European middle income country where maternal mortality was low but where women had poor access to pain relief during childbirth and limited use of regional anesthesia during cesarean section [14].

*“the regional anesthesia rate was <1 %, family members weren’t allowed in the delivery room and women endured the delivery alone, in pain and afraid.” – hospital anesthesiologist*

During the seminar, this anesthesiologist demonstrated interest and enthusiasm for Kybele’s work. She was encouraged to collect and analyze data on the use of obstetric regional anesthesia in Croatian hospitals. She was sponsored to spend three weeks at Wake Forest and Duke University Medical Centers in the United States gaining knowledge and expertise in conducting delivery with the assistance of epidural analgesia.

##### Stakeholder support

Upon her return to Croatia, she organized lectures for obstetricians at her hospital and formed a seven member team of anesthesiologists to conduct epidural pain relief. Courses for pregnant women were initiated and through the media, a message was sent that pain free delivery services were available. Two labor rooms were refurbished to enable a family member to participate in the delivery. She became a natural champion and an ideal candidate for a partnership.

##### Partner involvement – customized work planning

In September 2005, she organized the first Obstetric Anesthesia course in Croatia as a symposium and a practical educational event. Kybele lecturers worked and lived on site for 15 days. The two day symposium was attended by anesthesiologists from neighboring countries as well. The Kybele instructors from 6 countries worked in 9 hospitals across the country in operating and delivery rooms educating local anesthesiologists, obstetricians, and midwives, most of whom had never had the opportunity to be part of the international team. An average of ten anesthesiologists received hands-on training in each hospital.

*“Most of my colleagues had never seen or performed spinal anesthesia for cesarean section or epidural analgesia for delivery until then. You wonder why? Due to strong traditionalism and religious beliefs, it was commonly accepted that a woman should endure pain during delivery. Although many of my colleagues had good command over regional anesthesia techniques, they had never performed those in pregnant women.” - Champion*

##### Monitoring and evaluation

Subsequent to this visit, the Kybele team provided ongoing consultancy and support. The results are encouraging. In participating hospitals, regional anesthesia use for cesarean section increased from 20 % to 34 % in one year alone. Prior to the program, the sponsoring university hospital, one of the largest delivery units in the country, performed no spinal anesthetics for Cesarean section. Instead, women were given general anesthesia and had no recall of the birth of their baby. In 2014, 55 % of the cesarean sections were done using regional anesthesia. Epidural pain relief services have increased as well [14].

This example highlights the first principle of Kybele’s model, which is finding an engaged local champion. In this case, the program took off because of the pivotal role of the champion who took over the Croatian side of the project. In addition to the work at the hospital, she initiated the Club of Pregnant Women and Parents and a lecture series entitled, “Knowledge for Birth without Fear.” The club was founded as a non-profit voluntary association of mothers in 2004 and uses weekly lectures to increase the knowledge and skills of prospective parents and the quality of care provided by health professionals. Data collected over the past 10 years have seen a continuous increase in participant numbers, now totaling over 10,000, from 498 in 2004 to 1,180 in 2014.

#### Serbia

##### Engaged champion

The partnership between one of the largest obstetric hospitals in Serbia and Kybele began in September 2012. The initial contact was made by a Kybele team leader who originated from this country and observed a gap in obstetric anesthesia practices. However, initially, local physicians did not perceive a need for further education. As a result, initial progress in establishing relationships was slow and frustrating. Finally, an anesthesiologist in a regional hospital offered to organize the first obstetric anesthesia meeting and workshop. This was held in 2012 for 80 participants. Four Kybele team members provided lectures and hands-on training in the operating room, delivery, and neonatology departments. For the hands-on portion, all hospital anesthesia staff (9 specialists and 1 resident) received education, along with 8 residents from other nearby hospitals.

##### Stakeholder support

In order to broaden the initiative, the second meeting and workshop involved more local physicians as organizers and trainers. The course, held in September 2013, received accreditation from the country’s health institutions. Participants came primarily from the local region, with a few from surrounding countries. Kybele lecturers were joined by five lecturers from three university hospitals in the country. The second course was supported by external grants raised by the local hosts.

##### Partner involvement – developing and maintaining trust

In September 2014, the 3rd Annual School of Obstetric Anesthesia was held and was supported by the National Society of Anesthesiologists in the country. It was extremely successful with participants attending from three surrounding countries. Lectures were presented jointly by Kybele and local staff. For the hands-on portion, there were 10 local staffs, some of who served as instructors, and 32 participants from wider geographical regions of the country and surrounding countries. Interest was so great that some individuals were turned away. The increasing interest has led to expansion to two programs per year.

This case study illustrates the importance of several of the Kybele principles of Fig. 1. The program could not begin until a local champion was willing to sponsor the first event. But the spread and success of the program required both stakeholder support and partner involvement. While in this case the champion was necessary, stakeholder support and networking were also important for the partnership to expand.

#### Ghana

##### Finding an engaged champion

Kybele’s association with this country began in 2006, when several team members made trips to demonstrate anesthesia techniques and to conduct neonatal resuscitation training programs at several hospitals. During these visits, it became apparent that there was also a critical need to improve childbirth safety and that this could be only achieved through a long term partnership [15]. Several hospitals were considered for this partnership, and an urban referral hospital was selected based on interactions with the enthusiastic chief obstetrician in the hospital, who was motivated to create a platform for change to reduce maternal and newborn mortality [15]. This hospital was performing 8000–9000 deliveries a year, with 80 % of these mothers being referred from other institutions within and outside the region, many of whom are high risk.

##### Building stakeholder support

In January 2007, Kybele entered a 5-year agreement with this country’s Ministry of Health to establish an obstetric center of excellence with the goal of reducing by half the number of maternal and neonatal deaths. In a cost sharing arrangement, the Kybele team agreed to make three trips to the country a year, and the ministry agreed to pick up the costs for local accommodation, food and transport.

##### Partner involvement – customized work planning

In the first year of the agreement, the hospital’s systems and patient care processes were jointly evaluated by Kybele and the local champion. The champion, the chief obstetrician in the hospital, was then invited to visit three US-based institutions of key Kybele faculty. Many ideas and recommendations for improvement crystallized as a result of this visit. Specific areas for improvement were identified and a strategic template or “process map” was jointly constructed to help all staff realize what aspects of patient care and systems management needed to be addressed [1, 15]. The template identified the problems, recommended realistic solutions, and charted progress towards defined goals [1, 15].

##### Partner involvement – developing and maintaining trust

A primary activity of this partnership has been to build the capacity of the local staff to make the hospital the local hub for best practice in maternal and neonatal care. The partnership identified *clinical champions* in each department who are nurses and midwives responsible for the care for mothers and babies. These champions are trained to lead quality improvement efforts in their wards and to train staff both internally and at other hospitals on good clinical practice. In addition, selected nurse managers and doctors are trained in leadership techniques and in quality improvement to take on cross departmental projects. Several midwives and nurses have visited Kybele team member facilities in the US and the UK to learn about patient care from high resource settings. At the same time, medical, nursing, and public health students from Kybele’s American and European institutions learn about providing care in low resource contexts. At this point, the project is being scaled up to four other regional hospitals in the country. The training for the scale up will be jointly conducted by Kybele and local staff.

##### Monitoring and evaluation

Monitoring data collected by the clinical champions and Kybele team members is used to identify performance gaps and to tailor the training program to address those gaps. The clinical champions have been trained to utilize the well-known Plan/Do/Study/Act approach to quality improvement where data are used to test and implement successive cycles of small changes. As the partnership scales up to other regional hospitals, there is an effort under way to develop a standard set of maternal and neonatal indicators that tertiary hospitals in the country can adopt.

### Unsuccessful partnerships

Not all Kybele attempts to partner with host countries were successes. At least three Kybele partnership attempts did not go as planned. These partnership attempts failed because one or more the principles in Fig. 1 were not in place. In some cases, communications were not strong enough for a clear and common understanding of purpose. In others, it proved difficult to appoint a program champion who could recruit and engage other stakeholders. Additionally, partnerships failed where language barriers resulted in miscommunicated expectations resulting in the inability to create clear working arrangements. A lesson learned from these unsuccessful partnerships has been to know when it is not productive to try and force a partnership. If the champion cannot be appointed and the stakeholders are not engaged in the first few sets of meetings, there is little value in persisting.

## Discussion

A comparison of the country examples reinforces the importance of the four principles of partnership shown in Fig. 1. All successful partnerships share all four elements and all unsuccessful partnerships have a deficit of at least one principle. However, a key learning for Kybele was that even among the principles, there were some that were more important than others. Not surprisingly, the need for a champion and engagement of stakeholders are foundational principles without which no partnership can sustain. Even if it takes some time to formulate a work plan and to involve partners in a productive way, having a strong foundation can serve as a buffer against delays and setbacks. However, if work plans are launched without a strong partner or the involvement of stakeholders, any short term gains from the work inevitably diminish over time. In Ghana, Kybele has had several false starts and hospital partnerships that have seen limited progress, and in all cases, the primary reason has been inadequate championship and partner engagement.

Once the foundational elements are in place, the next most important element has been the collaborative design of a flexible and customized work plan. There are several factors that make this possible. The first is that the Kybele team has a large pool of professionals with diverse skills to choose from, both in terms of expertise and seniority. Depending on the particular nature of the partner’s needs, an appropriate team of senior and junior doctors, midwives, nurses, public health professionals and management and leadership experts can be assembled. The second factor is that Kybele’s team members are multinational and international in terms of nationality, geography and ethnicity, and this provides a base of diversity from which it possible to connect with partners from a variety of countries and regions.

In order to maintain this flexibility, and to attract and retain volunteers willing to be part of the Kybele team, Kybele uses its partnership principles to grow and sustain its own inter-organizational partnerships. As mentioned previously, every country has a team leader, who serves as the Kybele counterpart to the country champion. Just as the country champions develop relationships with in-country stakeholders, the team leaders recruit and manage the Kybele team members. Based on the work plan and the priorities, the team leaders are responsible for assembling the team that is most appropriate for the visit. The Kybele team leaders are also responsible for ongoing involvement of internal partners, and this is especially important because a large percentage of Kybele team members are volunteers. In order to keep them involved and enthusiastic, they need to be provided non-monetary incentives such as academic credit, faculty mentorship or global health experience, as well as increasing responsibility within the Kybele team. It is important to emphasize this as a key aspect of Kybele’s partnership approach. In order to build credible external partnerships, it is equally necessary to establish a credible internal base. This allows the Kybele team members to “lead from within” instead of just being seen as external experts.

## Conclusion

General principles of successful partnerships have to be adapted and interpreted to fit the context of each organization’s goals. This interpretation takes place over time as an organization develops its partnership and learns what works and what doesn’t in the organization’s unique context. For organizations such as Kybele, which work in a variety of diverse settings, it is even more important to get the partnership formula right. Over years of experience in many countries, the Kybele partnership model has distilled the six stages of successful partnerships described in the literature to four interrelated principles. These principles have been shown to be robust across all of Kybele’s global health programs.
